# Body contouring surgery in a massive weight loss patient: An overview

**Published:** 2008-10

**Authors:** Prabhat Shrivastava, Aditya Aggarwal, Rakesh Kumar Khazanchi

**Affiliations:** Department of Burns, Plastic, Maxillofacial and Microvascular Surgery, Lok Nayak Hospital and Associated Maulana Azad Medical College, New Delhi-110002, India; 1 Department of Plastic and Cosmetic Surgery, Sir Ganga Ram Hospital, New Delhi-110060, India

**Keywords:** Bariatric surgery, brachioplasty, lower body lift, massive weight loss, morbid obesity, thighplasty, upper body contouring

## Abstract

The number of patients with history of extreme overweight and massive weight loss (MWL) has risen significantly. Majority of patients are left with loose, ptotic skin envelopes, and oddly shaped protuberances, subsequent to weight loss. Redundant skin and fat can be seen anywhere on the body following MWL. This group of population presents many unique problems and challenges. Body contouring surgery after MWL is a new and exciting field in plastic surgery that is still evolving. Conventional approaches do not adequately cater to the needs of these patients. Complete history, detailed physical examination, clinical photographs and lab investigations help to plan the most appropriate procedure for the individual patient. Proper counseling and comprehensive informed consent for each procedure are mandatory. The meticulous and precise markings based on the procedure selected are the cornerstones to achieve the successful outcome. Lower body contouring should be performed first followed six months later by breast, lateral chest and arm procedures. Thighplasty is usually undertaken at the end. Body contouring operations are staged at few months' intervals and often result in long scars. Staging is important as each procedure can have positive impact on adjacent areas of the body. Secondary procedures are often required. However, proper planning should lead to fewer complications and improved aesthetic outcome and patient satisfaction.

## INTRODUCTION

In the past decade, there has been an alarming increase in the prevalence of obesity worldwide. Obesity has been identified by the World Health Organization as a “global epidemic”.[1] In the US, no state had obesity prevalence rate >20% in 1995; in contrast, by 2005, only 4 states had obesity prevalence rate of <20%. By current estimates, approximately 66% of United States adults are overweight or obese, of which 15 million are now categorized as morbidly obese. In India too, the changes in lifestyle, reduction of physical activity with consequent reduction in energy requirement is leading to increase in obesity. National Family Health Survey (NFHS-3) has shown high prevalence of obesity in urban areas in states of Punjab, Delhi and Kerala.[2] In recent years, improved surgical and conservative measures have become available for controlling obesity. Consequently, there has been an exponential growth in the number of patients presenting for body-contouring procedures following massive weight loss.

## OBESITY CLASSIFICATION

Body mass index (BMI) of an individual is calculated as weight in kilograms divided by the square of height in meters (kg/m^2^). American Society for Bariatric Surgery has classified obesity as per BMI values [Table 1]. BMI up to 24.9 is considered as normal. Overweight is defined as a BMI between 25.0 and 26.9 kg/m^2^, and extreme (morbid or class III) obesity is defined as BMI exceeding 40 kg/m^2^. Individuals with BMI >35 kg/m^2^ with major co-morbid condition(s) like obesity related hypertension, diabetes etc. are also classified as morbidly obese. It has been recognized that obesity is a chronic disease and any form of treatment aims at palliation rather than cure.[3]

**Table 1:** Obesity classification by American Society for Bariatric Surgery

*BMI value (kg/m^2^)*	*Category*
18.5-24.9	Normal
25.0-26.9	Overweight
27.0-29.9	Mild obesity
30.0-34.9	Moderate obesity- Class I
35.0-39.9	Severe obesity- Class II
40.0-49.9	Extreme (morbid) obesity- Class III
50.0-59.9	Super obesity
60.0 +	Super-super obesity

## WEIGHT REDUCTION STRATEGIES/MODALITIES

Although regulating dietary intake of calories (low calorie / very low calorie diets), regular exercise, behavior modifications, pharmacotherapy (fenfluramine, phentermine) and herbal medications can achieve modest weight reduction, statistics reveal a high incidence of relapse with >90% of such individuals regaining weight.[4,5] Since its inception in 1966, bariatric surgery continues to offer the greatest degree of sustained weight loss to the morbidly obese.[6,7] It has been acknowledged as the riskiest, but the most efficacious weight loss method. In the US, the number of bariatric procedures increased from 13,000 in 1998 to nearly 200,000 in 2006.[8]

Improved peri-operative and long-term care and refinements in technique have rendered bariatric surgery an increasingly safe and reliable method of weight loss. Bariatric surgery is also being performed in people who are severely obese (BMI 35° - 40°).[9] This upward trend has created a large population of post-bariatric weight loss patients who approach a normal BMI range, and they want to look as normal as possible.

## BARIATRIC SURGERY PROCEDURES

An understanding of various bariatric surgical procedures is important in evaluating the patient for body-contouring procedures. Three main categories of bariatric surgical procedures are: restrictive, malabsorptive and the restrictive-malabsorptive.

Purely restrictive procedures produce satiety by creating a small gastric pouch with a restricted outlet, and include horizontal gastroplasty, gastric partitioning, silastic ring gastroplasty, and the vertical banded gastroplasty (VBG). The Bioenterics LAP-BAND System (adjustable gastric banding), approved by FDA in 2001 has nearly replaced VBG as a purely restrictive procedure. The band is placed laproscopically and is considered as the least invasive of all the bariatric procedures. The band diameter can be changed by adjusting a balloon connected to a subcutaneously placed access port.

Malabsorptive procedures include jejunoileal bypass (JIB), biliopancreatic diversion (BPD), duodenal switch (DS) operation etc. Most of the nutrient absorptive surface of the gastrointestinal tract is bypassed by creating an anastomosis from the proximal to the distal small bowel. Although, these procedures achieve rapid and significant weight loss, they carry a high risk of long-term nutritional and metabolic complications. Therefore they are in limited use and typically are reserved for the patients with BMI > 50 kg/m^2^.

The most popular combination restrictive-malabsorptive procedure has been Roux-en-Y gastric bypass (RYGB). The weight loss in standard RYGB is primarily by restriction of food intake which results from the creation of small gastric pouch with a 1 cm outlet. Malabsorption with RYGB is due to the bypass of the fundus, duodenum, and the proximal jejunum. Laparoscopic RYGB and the Lap-band procedure have become the most commonly performed bariatric surgery operations today.

## CONTOUR DEFORMITIES AFTER MASSIVE WEIGHT LOSS [FIGURES 1A–H]

**Figure 1:**
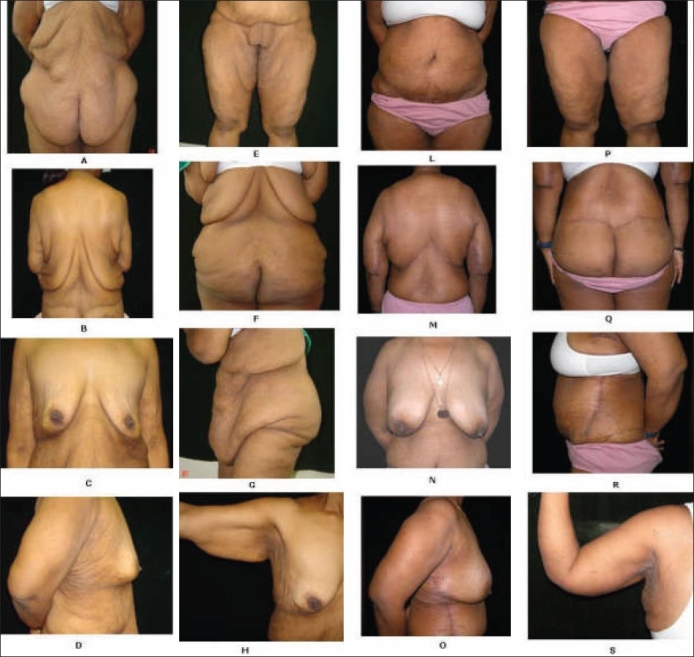
A 26-year-old female after weight loss of 90Kg by dieting and exercise. Figs. 1A to H are preoperative pictures showing contour deformities after massive weight loss. Figs. 1L to S are pictures showing the result after five body contouring procedures which included a circumferential belt lipectomy, upper body contouring, augmentation mastopexy, lateral thoracic and abdominal excisions, brachioplasty and thighplasty

Massive weight loss (MWL) is defined as 50% or greater loss of the *excess* weight.[10] The contour deformities after bariatric weight loss encompass diverse and unexpected manifestations that potentially involve every area of the body. After a rapid and massive weight loss, there is a sudden change in BMI which leads to skin and soft tissue excess and poor skin tone. There is often a *‘deflated appearance’* more pronounced in the breasts, buttocks and the face. The skin and the soft tissues fail to retract completely and become redundant, collapsing inferiorly and inferomedially from the characteristic areas of fat deposition. *In the upper trunk*, the redundant tissues from the axilla and flank contribute to the upper and mid back rolls and flank rolls. There occurs varying degrees of breast ptosis and excessive skin under the upper arms. *In the lower trunk*, the redundant tissues of the lower abdomen and the pubic area fall directly towards the inner thighs. There can be enormous overhanging pannus that disrupts the silhouette. The collapse of the redundant tissues from the lower abdomen, mons pubis, buttocks as well as from the medial thigh itself contribute directly to the excess tissues along the thighs resulting in both a vertical and horizontal tissue excess. Striae are present throughout the torso. Moreover, women tend to have large amounts of cellulites as well, particularly along the hip region. Often there is pain, irritation and intertrigo under the massive skin folds.

### Pittsburgh rating scale

Song *et al*,[11] have designed an all inclusive and illustrative classification system that helps in systematically assessing and quantifying the level of deformities in each particular region. Ten anatomical areas delineated for analysis include arms, breasts, abdomen, flank, mons, back, buttocks, medial thigh, hips/lateral thighs, and lower thighs/ knees. Because of their complexity, face and neck regions have been excluded. A four point grading scale is designed for each region: grade 0- normal range, grade 1-mild deformity, grade 2-moderate deformity, and grade 3-severe deformity. Generally, a mild deformity would require non-excisional or a minimally invasive procedure; a moderate one would need an excisional procedure while a severe deformity would require combinations of excision, lifting and would involve large areas of undermining.

The Pittsburgh rating facilitates the preoperative planning and is a useful tool in quantifying the improvement in appearance attributable to surgical manipulation.

## PREOPERATIVE WORKUP OF A PATIENT WITH MWL

### Clinical and lab evaluation

A comprehensive preoperative evaluation is mandatory because the body-contouring procedures following MWL are often extensive with the potential for significant morbidity and even mortality.[12,13] The emphasis should be on several areas: weight loss history, type of bariatric procedure done, diet and exercise habits, residual medical problems, co-morbid states, physical examination and laboratory studies. A Plastic surgeon must ensure that the patient is *weight stable* (not more than 1 to 2 lbs per month fluctuation over 3-6 months, before surgery) at the time of undergoing body contouring. Usually, weight loss stabilizes within 18-24 months following bariatric surgery. If the body contouring procedures are performed on a patient with ongoing weight loss, there would definitely be early recurrence of tissue laxity.

Besides evaluating the deformities of aesthetic and functional concern, incisional hernia(s) and features of nutritional deficiency, if any, should be looked for and documented. Areas of adhesion where overlying skin and soft tissues do not slide, should be noted. The flap perfusion past these lines of adhesion can jeopardize the flap circulation. For lap-band patients, the access port should be palpated and its location recorded as it may require relocation on the abdominal wall during an abdominoplasty operation. The lab tests should be done at least 4 weeks prior to the surgery to allow for enough time to address and correct any deficiencies. The list should include CBC, serum electrolytes, prothrombin time, partial thromboplastin time, serum albumin and per-albumin levels, ECG and chest radiograph. Besides these, the values for micronutrients (iron, vitamin B_1_ and B_12_, folate, calcium, and vitamin D) may also be obtained guided by history, physical examination and type of bariatric procedure.

A written clearance should always be obtained from cardiologist, pulmonary physician and psychiatrist. Patients who are dissatisfied with their postoperative results following body contouring have used their preoperative psychiatric history as a part of their legal action against the plastic surgeon, arguing that their psychiatric condition prevented them from fully understanding the procedure and its potential outcomes. It is strongly recommended that plastic surgeons must obtain, preoperatively, a written confirmation from the treating psychiatrist that the patient is psychiatrically stable. This would offer protection to the surgeon in such adversities.

Pre-anaesthetic checkup should be done by a senior anaesthetist preferably with experience in managing MWL patients. Other areas of concern which should be discussed in detail with the patient include motivations and expectations, appearance and body image concerns.

### Informed consent

Many patients who have lost massive amounts of weight hold unrealistic expectations that the body contouring surgery will result in a total body transformation. Such patients are more likely to express disappointment and dissatisfaction with their postoperative result. The plastic surgeon should make the patient understand that body-contouring surgery produces large and visible scars. It is vital to review the exact placement of these scars with the patient. The patient must understand that visible scars are necessary for improved appearance in clothing. There is definite possibility of skin irregularities and residual deformities in body shape. Surgery may improve body contours; it will not result in a “perfect” body shape.

The patients must understand that how-so-ever tight the skin may be pulled, over time there will be a degree of relaxation which will contribute to *‘scar migration’* and some loss of contours. To some degree, relapse is expected. Secondary procedures in MWL patients are common and the revision policy should be made clear to the patient. In addition, the insurance concerns and the possible complications like seroma, hematoma, infection, dehiscence, lymphoedema, asymmetry, contour irregularities, unacceptable appearance /loss of umbilicus, vulvar distortion, sensory / motor loss, deep vein thrombosis, cardiac and pulmonary complications, detrimental effects of smoking on wound healing etc. should also be discussed with the patient.

Patients also need to be counseled that body contouring surgery after MWL is a process rather than a one time event, and hence multiple, often staged procedures may be necessary to meet their goals, each of which would encompass further risk, recovery time and often expense. Family members should also be fully informed as to the risks of surgery. It is helpful to have a photo album of previous successful cases to demonstrate what the patient can anticipate immediately postoperatively as well as in the long-term.

### Scar placements

Patients who have experienced massive weight loss following bariatric surgery develop myriad of contour deformities all over the body. Most would readily accept surgical scars in any location for improved contours. However, proper selection of the operative procedure and meticulous markings are immensely helpful in ensuring best placement of scars. The location and extent of any previous scars, the need for subsequent procedures at a later date and the degree of tissue laxity above and below the proposed scars must always be kept in mind while planning the incisions. An open cholecystectomy subcostal scar is a contraindication to extensive flap undermining. The vertical midline scar of open gastric bypass should preferably be incorporated in the operative design. The situation and length of the scars should be clearly discussed with the patient during consultation.

### Markings and photographs

**Figure 2:**
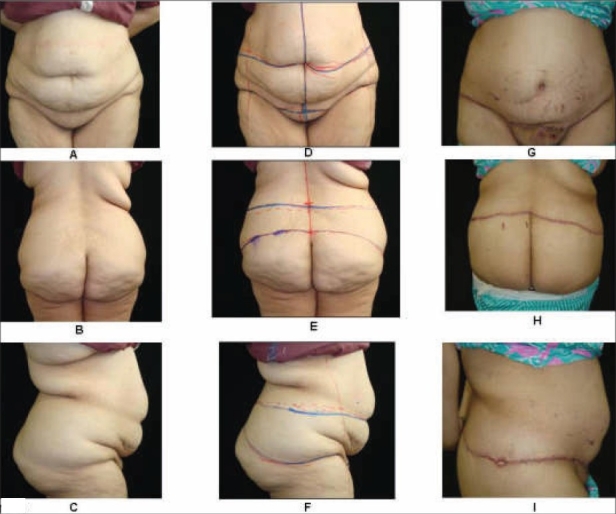
A 22-year-old female following 55 Kg weight loss after gastric bypass surgery. Figs 2A to C are preoperative pictures of contour deformities. Figs 2D – F show preoperative markings and prior to belt lipectomy. Figs 2G to I are early post-operative pictures after lower body contouring

As with all body contouring operations, the markings form the cornerstone of a successful outcome. Meticulous and precise markings should be done for the selected procedure at least one day prior to the scheduled date for surgery, and the patient should be photographed in all the standard views after the markings. This provides the surgeon an opportunity to evaluate the photographs to reassess the deformities. Suitable adjustments should be made, if need be, the next day during surgery. Hatch marks should always be made above and below the incision lines to bring the tissues in proper alignments at the time of closure. The similar views of the patient should be taken post-operatively (immediate and long-term post-op) with same background and illumination. Such albums are very useful and handy for education of the patient and the plastic surgeons as well [Figures 1 and 2].

### Role of liposuction

There are differing schools of thought on the role and staging of liposuction in MWL patients. Some surgeons prefer to perform *simultaneous liposuction* arguing that it allows one area to be treated and rejuvenated in one sitting while eliminating the need for extra surgery. However, significant oedema caused by the procedure can not only reduce the vascularity of the flaps, it may even compromise the final outcome. Others opt to perform liposuction six months prior to the excision. Debulking of the area is thus achieved and the potential risks to the flaps due to resultant oedema are also eliminated. The demerits are an extra surgical stage, hospitalization, increased costs and additional recovery time. In addition, the tissues may be stiffer making the later flap advancement more problematic.

## LOWER BODY CONTOURING

### Goals

The objectives of lower body contouring include flattening of the abdomen, excision of the lower back rolls, relocation/recreation of the umbilicus, creating a waist line in women, reshaping/redefining the buttocks, lifting the antero-lateral thigh and elevation and/or reduction of the mons.

### Surgical options

As the deformities after MWL are circumferential in nature, the treatment should also be circumferential to address the trunk as a unit.[14] Most of the MWL patients seeking improvement of their lower body would require a *circumferential* body lift along with gluteal contouring, mons reduction, excision of the flanks and a thigh lift. Anterior resection only, may be chosen as part of a staged procedure or when the circumferential treatment is not an option. Conventional thigh lift, buttock lift, abdominoplasty etc. fall short of achieving the optimal outcome and are often stretched to their limits in MWL patients. Attempts to manage the patient who has undergone bariatric surgery with abdominoplasty and liposuction alone are likely to result in an unsatisfactory outcome. Unsightly scars, ‘dog-ears’ or flattening of the body curves occur. Due to these limitations, various techniques have been described to treat post-bariatric lower abdomen circumferentially. These include circumferential belt lipectomy,[15,16] circumferential torsoplasty,[17] lower body lift[18] and body lift.[19] Although they have different names, each involves a simultaneous abdominoplasty and a thigh and buttock lift. The main disadvantages of circumferential procedure are that the operating time is prolonged as the tissue resection and the incision length are doubled, and the patient needs to be repositioned intraoperatively. If increased surgical time or patients' medical condition poses a significant risk, the procedure should be staged into an anterior dermolipectomy followed by a buttock lift/gluteal contouring and thigh lift at a later date.

Circumferential abdominal lipectomy is an extended abdominoplasty operation without thighs and buttocks undermining. The incision course extends into the back and the buttock regions on both sides. This serves solely to prevent dog ears and doesn't produce any tightening effects on the back or buttocks. There is much scarring with unsatisfactory result especially in the back areas.

Circumferential belt lipectomy is relatively more extensive operation wherein excision of the excess and redundant tissue is performed circumferentially directly at the hip, back and anterior abdominal region.[15,16] The excised wedge is more extensive and broader. The operation is performed with the patient first in supine and then in both lateral decubitus positions. Although it results in a relatively high circumferential scar above the iliac crest, the tightening effect at the back particularly in the waist region is far superior compared to the circumferential abdominal lipectomy. It creates a more defined waist and has lesser impact on the thighs.

Lower body lift involves more extensive undermining and aggressive resection.[18] The operation is done with the patient in prone and then the supine position. Discontinuous cannula undermining and adjunctive ultrasound assisted liposuction of back, hips, sides and epigastrium are added to provide better contours. It stresses the importance of meticulous handling of superficial fascial system (SFS) and approximating the SFS with permanent sutures to maintain the soft-tissue contours and to maximize the scar quality over the long-term.[20–22] The dissection is done *below the Scarpa fascia* resulting in resection of complete superficial soft-tissue layer. The volume reduction thus achieved may result in flattening of the buttocks.

In circumferential suprafascial lower truncal dermolipectomy[23] the dissection is done *above the Scarpa's fascia* (dorsally as well as ventrally).The fascia remains attached to the deep fat in the resected areas circumferentially. The resilient fascia can be used for repositioning and remodelling in different vectors. The stretching of the superficial fascia system occurs in the vertical-medial direction independently of the vector along which the skin is stretched. Gluteal superficial fascia system tightening in the buttocks absorbs much of the tension. This has been referred to as “gluteal SMAS” analogous to the facial SMAS in face lift surgeries. In the area of the abdomen, strong pulling of the fascial system achieves an additional lifting effect on the thighs and mons pubis.

### Marking [Figure 2D–F]

Precise pre-operative marking is essential for a successful outcome. Markings are done with the patient in standing position. Besides marking the anterior (from xiphoid to anterior vulvar commissure) and posterior vertical midline, additional full length paramedian vertical reference lines should be drawn symmetrically on either side to provide exact orientation during surgery. Loose, redundant skin and subcutaneous tissues fall to one side in supine/lateral position and the orientation is further lost once the flaps are elevated. The folds of redundant tissues make it difficult to identify the anatomical bony landmarks used for achieving symmetry. The ptotic tissues must be lifted against the gravity while transverse incisions are marked. It is recommended to do the markings with the patient slightly flexed at the waist. This simulates the position the patient will assume after completion of the anterior resection. Failure to mark in this position is likely to put undue tension on the suture lines, especially on the dorsum which may lead to wound dehiscence postoperatively.

Posterior markings are done first. The upper incision lines on either side follow the buttocks subunit in a gentle convexity at the level of the posterior iliac crest. From either side, the lines dip slightly towards the midline, angled towards the gluteal furrow, in a shallow V. This provides an optical accentuation of the buttock form. The degree of tissue laxity above the incision also needs to be considered when determining the uppermost point of excision. To determine the *lower incision line*, the buttock and the lateral thigh are serially mobilized up to the superior level mark using the pinch test. The upper incision shall remain relatively fixed with maximum mobilization coming from below. The lower incision is marked in the form of a “lazy S”. Hatch marks are also added to act as useful reference points during surgery as well as to helping proper alignment of the flaps at the time of closure.

Anterior marking is begun by vertically lifting the mons with one hand and drawing the horizontal incision within the pubic hairline, about 5-7 cm above the upper vulvar commissure (or base of the penis) with the other hand. Tissues in the inguinal region are next lifted supero-medially. The incision line from the pubic tubercle (the lateral edge of the pubic hair) ascends obliquely supero-laterally towards the anterior superior iliac spine to join with the previously marked posterior lower incision line on both the sides. The pinch test is once again done serially to determine the superior incision line for anterior resection, in much the same manner as in abdominoplasty. This level may go above the umbilicus.

### Position and technique

Some surgeons prefer to operate with the patient in prone to supine position, while others favour supine to lateral decubitus position. The patient is circumferentially prepped with the povidone iodine from shoulders to the ankles in the standing position. The patient is then draped with a sterile half sheet and asked to lie on a sterilely draped operating table. Sterile stockings and sterile sequential compression devices are placed. A Foley's catheter is placed following general anaesthesia. The large excess of tissues can get compressed and lead to necroses. In prone position in particular, pressure necrosis can form around the periorbital region and the breasts. Arms also need to be sufficiently padded to prevent neuropraxia. The entire team must bear these considerations in mind while positioning the patient.

When prone to supine position is used, full thickness cephalad incision is made first through the skin and subcutaneous fat down to the level of the deep fascia. Undermining is done at this level towards the marked inferior incision taking care to maintain a fatty layer of tissue overlying the deep fascia. This helps in preserving the lymphatic network and reduces the risk for seroma formation. The lateral thigh is mobilized either bluntly or with liposuction cannula. The flap thus raised is pulled up to confirm the lower incision markings and resection is accomplished. Suction drains are placed and closure is done in three layers. Three point sutures are taken to secure the superficial to the deep fascia to obliterate the dead space. Number 1 polyglactin is used to approximate the superficial fascial system and deep dermis while number 0 polyglactin is placed at the level of dermis. Because of the wound length, 3-0 running poliglecaprone is used for subcuticular closure thus obviating the need for suture removal. This layered closure minimizes the tension along the suture lines during the early months of scar maturation. Many surgeons in addition use topical skin adhesive for final cuticular approximation.

After closure, the lateral aspects on either side are left with big dog ears. These will need to be taken out with the anterior resection. The patient is turned supine and the anterior resection is accomplished similar to an abdominoplasty with the incisions joining the posterior incisions. While taking out the skin laterally, the thigh should be abducted to maximize the excision. Throughout the procedure the subcutaneous tissues are divided and the flaps are elevated with cautery set to a high level. The anterior abdominal wall flap is elevated to the level of the umbilicus which is preserved as usual. Superior to the umbilicus, the dissection is kept over the rectus abdominis muscles to the level of the xiphoid. The anterior abdominal wall flap is divided in the midline to the level of the umbilicus to facilitate exposure of the xiphoid region.

In patients who have had a lap-band procedure, the ports are often secured in the paramedian position in the anterior abdominal wall fascia. It may get buried and rendered inaccessible with routine plication. At this stage, the port should be moved to the subcostal position. Plication is then performed making sure that the port tube has not got entrapped in any suture. After the plication is complete the port should be secured to the fascia in the subcostal position. The two layer fascial plication is done with Number 1 polypropylene suture. Haemostasis is secured and layered closure is done over suction drains brought out through mons pubis. Following extubation, the patient is transferred to either ICU or ward in a beach chair position.

In the supine to lateral decubitus position, the anterior abdominal resection is done first. The umbilicus inset is completed, drains placed and complete closure done (as above), up to the lateral border. A large dog ear on both sides is temporarily closed with staples and the patient is turned to left lateral decubitus position with waist flexed to approximately 30° and knees to 45°. The entire hip roll region is removed and the dog ear excised after undermining the lateral thighs. Once again, the legs should be kept abducted to maximize the lateral ‘take-out’. Similar procedure is then done on the other side with patient in right lateral decubitus position.

### Post-operative care

Patient is restricted to the bed for first 24h. Thrombosis prophylaxis is given using low molecular weight heparin. Assisted ambulation is encouraged from Day 2. Sequential compression devices and Foley's catheter are removed if the patient is ambulating well. Hb%, PCV, serum electrolytes and blood sugar are monitored. PCA (patient controlled analgesia) pump is given, if available. Broad spectrum antibiotics and anti-inflammatory analgesics are continued for five days. Drains with output of less than 30 ml in past 24h are removed after five days. However, all drains should be removed by five weeks regardless of the output. Before discharge, the patient is fitted with compression garments to be worn for six-eight weeks.

### Gluteal contouring

Significant adipose tissue loss in buttocks results in ptosis and decreased projection leading to varying degrees of platypygia. In addition to the skin laxity and the volume loss, relaxation of the superficial fascial apron contributes to gluteal ptosis. Aggressive lifting performed to improve the contours of the thighs and lower back in circumferential body lifts can further exacerbate platypygia which results in further flattening. Gluteal aesthetics in a MWL patient can be enhanced with autologous tissue augmentation, large volume autologous fat transfer or alloplastic implants. Adjunctive techniques such as resection and tightening of SFS (gluteal SMAS), posterior thigh lift, infragluteal diamond lift can refine the results.

### Autologous gluteal augmentation (AGA)

Gluteal auto augmentation can be accomplished using either bilateral de-epithelialised ‘island AGA flaps’, a ‘moustache AGA flap’[24] or ‘superior gluteal artery perforator flaps’.[25] The flaps are designed and moulded using autologous skin and fat that would otherwise be discarded in the posterior portion of lower body lift.

Island AGA flaps are outlined as two separate islands, one on each buttock and de-epithelialised. The dissection is bevelled down through the SFS and the gluteal fascia to create two dermal islands. Muscle fascia is released on the superior and lateral aspects of the flap to increase the mobility of the islands. After confirming the position of the maximum projection point, the body-lift inferior skin flap is advanced over the island AGA flaps. The dead space may be reduced by placing sutures between the overlying body-lift flap and the island AGA flaps. The SFS is sutured, drains placed and the closure is done in layers. Although reasonable results are obtained, the amount of volume that is produced is insufficient to overcome the gluteal flatness in most MWL patients. Moreover, the point of maximum projection is higher than ideal.

Moustache AGA flap design is the modification of the island AGA flaps with placement of the central bridge of tissue and lateral flap extensions or handlebars. It is a partial island and partial transposition flap. The moustache handle bars are elevated from the fascia and rotated inferomedially. This recruits additional tissue for augmentation and lowers the point of maximum gluteal projection to the level of mons pubis. Additionally flap is imbricated to itself laterally to prevent trochanteric fullness. Moustache flap is indicated when a reasonable augmentation is desired especially for female patients.

Superior gluteal artery perforator flap for gluteal auto augmentation is a large oval-shaped de-epithelialised flap based on the previously dopplered SGA perforators, dissected from lateral to the medial aspect till the lateral perforator is encountered approximately 9 cm from midline. The flaps are rotated inferomedially, placed in a previously created gluteal pocket and tacked to the gluteal fascia. The closure is done in the same way as for the island AGA flaps.

### Autologous fat transfer

Large volume autologous fat transfer can be used in select patients as an adjunctive mode to enhance the buttock shape. Its efficacy as a primary modality in MWL patients is contested vigorously in literature. Many MWL patients do not have adequate donor sites for fat harvest. Nonetheless, it is clinically effective and can play a vital role in body contouring.

### Alloplastic implant augmentation

MWL patients do not have sufficient pad of fat to cover the implant, and their skin is thin which makes them susceptible to implant visibility, palpability, migration and extrusion. Moreover, the implant designs have limitations to be used as primary modality for gluteal augmentation in pronounced platypygia.

### Monsplasty

The mons is almost always ptotic and may exhibit both horizontal and vertical excess. It may be partially or totally hidden under the overhanging anterior abdominal wall pannus. Mons should be rejuvenated as a part of the abdominoplasty / body lift procedure. The vertical mons excess can be excised by placing the lower transverse incision for body lift 2 cm below the pubic hairline (about 5-7 cm above the superior vulvar commissure-at the level of the pubic bone). Too low incision would interfere with the lymph drainage and innervation. Vertical, wedge excision may be incorporated to reduce the mons width. The ptotic mons tissue should always be resuspended to the superficial fascia of the abdominal flap. Care should be taken not to pull the mons too high, lest it may alter the position of the clitoris and / or urethral meatus. Mons reduction surgery can be scheduled at a later stage also along with other additional procedures.

### Umbilicoplasty

The final appearance and location of the umbilicus on the anterior abdominal wall is often viewed by patients as an important measure of success. Smaller umbilicus is aesthetically more pleasing than the larger umbilicus. Many techniques have been described to preserve or recreate the aesthetic appearance of umbilicus. Several incisions (horizontal crescent shaped, inverted V incision, single vertical incision etc.) have been employed with the aim of making an opening of sufficient size and achieve some superior hooding. Concentric circles should be avoided as these rings exhibit contraction later distorting the shape. It results in an abnormally small umbilicus which is not only unnatural but presents hygienic problems also. In patients who have had umbilical hernia repair or vertical midline hernia repair done earlier, viability of the umbilicus becomes doubtful. In such cases, neo-umbilicus reconstruction needs to be performed either primarily or at a later date to achieve a pleasing outcome.

## UPPER BODY CONTOURING

The regions included in the evaluation of upper body are the breasts, lateral chest, arms, upper and mid back. The plastic surgeon must remember that the circumferential lower body lift operation exerts a significant positive impact on breasts, flanks and upper back. It reduces the magnitude of the upper body contouring procedures required or may occasionally even eliminate their need. However, in women it can also result in significant downwards migration of the inframammary folds. Precisely for this reason, it is recommended not to perform the breast contouring surgery before or even with a body lift operation.

The upper arms, lateral chest and the breasts are intimately related and all need contouring usually in one stage for adequate rejuvenation of the area. The location and direction of lateral inframammary crease in both women and men is assessed. If the patient has a normal upward sweeping lateral inframammary crease, he / she would present with breast and upper arm deformities that are independent of each other and can be treated by independent breast reconstruction and brachioplasty procedures. However, if lateral crease position is lower, the breast, arm and lateral chest deformities may be treated simultaneously. Thorax then is treated as a unit. However, the staging sequence is surgeon and patient-specific.

The goals to be achieved are to restore projection and fullness of breast parenchyma with appropriate NAC size and position, to reposition / recreate the inframammary fold, to eliminate axillary skin rolls, mid and upper back rolls, and to reshape the arms by excising loose, hanging and redundant folds of excess skin and fat.

## Breast reshaping

In MWL patients, loss of breast parenchyma and glandular tissue results in marked breast volume deflation with flattening, while loss of skin tone and elasticity leads to severe ptosis. The nipple position is more medialised and the volume loss may be asymmetric. Additionally, axillary skin rolls and lateral breast rolls blur the borders between the lateral breast and the chest wall. The lateral breast rolls often continue posteriorly as upper back rolls. There is descent of lateral inframammary crease to varying degrees. In view of these extensive deformities, traditional mastopexy procedures are often inadequate in a MWL breast.

Some surgeons elect to perform an augmentation-mastopexy in single stage, while majority prefer to perform mastopexy alone first followed by augmentation at a later date if a larger size is desired. Combining augmentation and mastopexy with transposition of NAC reduces vascularity of the flaps and may compromise healing. Moreover, the implant position is difficult to control, and malposition with descent are common complications. The submuscular / subglandular placement of the implant is dictated as per the preference of the surgeon and the patient. Smaller implants are preferable as the heavier implants might contribute to the risk of recurrent ptosis and scar-widening. The combined procedure requires experience and expertise, and secondary procedures are often needed to optimize the outcomes.

Regardless of the technique used for skin resection, aesthetic result should achieve bilateral symmetry, good projection, superior fullness, correctly positioned NAC, with well defined lateral curvature and inframammary fold. The normal inframammary fold has a semicircular shape, with its lateral aspect rising superiorly as the lateral chest wall is approached. It may be necessary to augment one breast and reduce the other to obtain symmetry. It is vital to make the patient sit up during the surgery to review various landmarks to obtain adequate symmetry.

For patients with severe ptosis and flattening of the breast shape, auto augmentation mastopexy has been described using a lateral deepithelialised dermal/breast parenchymal flap.[26] This selectively adds tissue to the breast mound, simultaneously eliminating the lateral skin roll deficiency. The central breast tissue is suspended to the rib periosteum to achieve superior pole fullness and long lasting results. Suspension of breast parenchyma to pectoral fascia has also been used.[27,28] However, recurrence of ptosis is less likely with suspension to rib periosteum. The only drawbacks of this technique are the need for intraoperative tailoring and the lengthy scars.

Autoaugmentation mastopexy in MWL breast has also been reported with cephalic rotation advancement of deepithelialised extended superomedial pedicle, incorporating the lower pole of the breast.[29] Glandular shaping is done through plication and the pedicle is suspended as needed to fill the upper pole. This provides an internal sling, narrows the wide breast and redefines the inframammary fold. Good pillar closure helps support the breast and its new shape. Intercostal artery perforator flap from the lateral chest has also been used for autologous augmentation mastopexy in a MWL breast.[30]

### Male breast rejuvenation

After MWL, due to excess skin and inelasticity, there is varying degree of breast ptosis along with the lateral rolls on either side. There is loss of definition of infra-mammary crease and, in some cases, excessive breast projection due to hypertrophy. The critical components of male breast rejuvenation are reduction in the bulk and projection, proper positioning of the nipple-areola complex and restoration of the natural appearing inframammary crease. In males, the NAC lies immediately lateral to the breast meridian and is closer to the infra-mammary crease. It is vital to explain to the patient the placement and extent of the scars in order to achieve the desired result.

It should be remembered that lower body lift operation in combination with liposuction often results in sufficient tightening of the flank and chest obviating the need for further surgery for upper torso. Additional liposuction may be needed in cases of mild residual ptosis. The excess tissues along the axilla and the lateral chest may be resected by designing a vertically oriented ellipse laterally in order to avoid more visible scars on the anterior chest. Care should be taken to avoid displacement of the NAC complex laterally. In severe ptosis, an inverted–T mastopexy with extension towards the flank gives good results. Bilateral flankplasty with *direct excision* of the excess hanging tissues at the level of inframammary crease is an alternative. However, in this procedure, a free nipple graft would be needed and it would result in a long scar along the anterior chest.

### Lateral thoracic excisions

The lateral chest wall excision should be performed immediately after the breast procedure is completed. Wearing a brassiere makes the redundancy more evident, with the excess skin and fat bulging over the top. These rolls can be removed either by *transverse excision* or by using *lateral excision* approach. Both approaches could affect the vascularity of the abdominal flaps; hence the lateral thoracic excisions should not be combined with the lower body lift operation.

### Transverse excision

While planning the transverse back excision, pinch technique is used sequentially to ascertain and mark the extent of the resection. It is helpful to mark the outline of the patient's brassiere along the back. This helps to place the intended line of closure between the upper and the lower outline of the brassiere. Crosshatch marks are made across the excision markings to achieve proper alignment during closure. The superior extent of the upper back rolls is incised first down to the level of the muscle fascia. The inferiorly based skin-fat flap is elevated to the proposed inferior level of resection. With the flap elevated superiorly, while the shoulder is pushed inferiorly, the flap is pulled in the ‘pants over vests’ fashion and the excess is tailored to the superior line of excision. Closure is done in two layers over the suction drains brought out laterally on either side. The lateral breast and upper back rolls thus are eliminated while the lateral inframammary crease also gets elevated to its proper position.

### Lateral chest wall excision

Most of the horizontal thoracic excess can be addressed by an elliptical excision extending vertically from the axilla to the inframammary crease, either independently or as a part of an extended brachioplasty. The lateral thoracic excision can even be extended down along the lateral abdominal wall to address the horizontal excess of the abdominal wall as well as the back laxity. However, the patient will then have a long scar laterally [Figure 1 O and R].

The technique basically is a wedge excision of the loose tissue. As it does not include undermining, if the markings are properly done, there is little chance of any skin loss. The markings are done with the patient in standing position. The lateral extent of the breast and the inframammary fold is marked as the lowest point of the proposed excision. The excess skin is pinched in the superomedial direction to determine the excess and the elliptical excision is marked. The posterior skin is less mobile as compared to the anterior breast skin. The centre of the posterior incision can be extended more posteriorly to maximize the resection in the mid-back region. However, this would result in a longer posterior limb which would need to be adjusted during closure. The final scar would lie well hidden under the arm when it is at rest. They are particularly helpful when the posterior flap needs to pulled superomedially to correct the lateral inframammary fold descent.

The anterior incision is made first down to the deep fascia and the flap is raised till the posterior incision mark is reached. The excess tissue is resected after the confirming that a safe tension-free closure can be accomplished. If required, the posterior incision marking can be adjusted during surgery. A lateral chest wall excision can not only elevate the inframammary fold, it can simultaneously reposition the lateral aspect of the breast. The breast may appear rejuvenated without any direct surgical intervention. The lateral chest wall excess has also been used for a modest auto-augmentation of the breast, which may be preferable by some to prosthesis.

### Brachioplasty

In MWL patients, excess sagging skin and fat generally presents around the arms in between the axilla and the elbow. The deformity occasionally extends distally onto the forearm, but invariably crosses from the arm to the axilla at the posterior axillary fold and onto the chest and the lateral breast. This distribution makes it nearly impossible to address one region without assessing its resultant effect on the adjacent areas. Liposuction alone is rarely sufficient to provide the aesthetic result. It needs to be decided after clinical examination whether to directly perform a resection or to first deflate the significantly over-inflated arms by initial liposuction prior to performing an excisional procedure 6 months later. Most patients would require a brachioplasty to achieve the desired results. Brachioplasty removes the excess upper arm skin and fat for aesthetic reshaping. Often the incisions are extended onto the lateral chest wall proximally and to the level of the elbow distally. The brachioplasty scars can be wide, long and often stay thick for many months. The short scar techniques are inadequate for correction of the deformities of the arms in a MWL patient.[31–33]

### Traditional excision

Excision by traditional T-type incisions is usually not sufficient to adequately remove the excess from the axilla without making the scars that are too visible. Incisions for the elliptical excision are made with the patient sitting with arms abducted to 90°. The anterior incision is made first up to the deep fascia and the flap is undermined till the posterior mark is reached. The medial antebrachial cutaneous nerve and the basilic vein should be carefully preserved in the distal half. After the excision is complete the wound is closed in layers over the suction drain. A Z-plasty should be incorporated if the excision crosses the axilla to prevent scar contracture and banding across the axilla.

### Double- ellipse marking and segmental resection-closure technique.[34]

Two ellipses are marked in this technique, one inside the other. The axillary crease level is identified and marked by abducting and adducting the patients arm. Starting from the axillary crease, with the arm abducted to 90°, the pinch technique is used to make the anterior and the posterior marks at each point along the upper arm. Joining these points anteriorly and posteriorly makes the outer ellipse. If the thoracic rejuvenation is to be performed simultaneously, the markings are continued onto the lateral chest wall along the posterior axillary fold dictated by the amount of excess. Distally the marks can cross the elbow if necessary. Excision along the outer ellipse would probably result in an inability to close the defect due to failure to account for the gap because of the fat in between the pinching fingers. Thus, an adjustment is made along each point along the entire upper arm. The inner ellipse is thus marked adjusted to the first ellipse based on the thickness of the pinch. For example if the distance between the pinching fingers is 2 cm, then the new ellipse marks are moved in 1 cm on each side. This adjustment need not be made onto the chest wall as the tissues can be suitably undermined on the chest. Crosshatch marks are made and a central line is drawn from the axilla to the end of the ellipse.

A sequential *‘segmental-resection-closure’ technique* is used to eliminate the possibility of being unable to close the arm because of intraoperative edema developing in the tissues left open for extended periods of time. Excision is started along the inner ellipse from the most distal aspect. The dissection plane is just above the muscle fascia. Once the first cross hatch mark is reached, the area of resection is temporarily closed with staples. This prevents the development of the significant oedema in that segment. The process is then repeated to the next cross hatch mark. A Z-plasty is performed at axillary crease level. The closure is done in two layers over the suction drain. Extremity should be kept elevated with elbow flexed to minimize oedema formation in the immediate postop period. Complications include wound dehiscence, infection, haematoma, seroma, lymphocele, sensory loss, neuroma and nerve compression (usually in ulnar nerve distribution). Compression garments should be advised only after two weeks.

### L brachioplasty

The L brachioplasty involves a continuous excision of excess skin from the arm through the axilla and onto the chest in the form of the inverted ‘L’.[35] The ‘L’ represents the shape of the excision, with the long limb along the medial axis of the upper arm, the short limb meeting at a right angle across the axilla along the mid lateral chest. The short limb scar of the L is hidden behind the anterior axillary fold. An inferiorly based triangular flap of the proximal posterior upper arm is advanced across the axilla towards the deltopectoral groove. This elevates the ptotic posterior axillary fold with tapering of the arm skin toward the axilla.

## MEDIAL THIGHPLASTY

Although major advances have been made in upper extremity and upper and lower truncal contouring, the thigh area continues to remain the most difficult and troublesome region to contour in MWL patients. It is recommended to perform thighplasty six months after the body lift operation. Lower body lift procedures have beneficial effect on the lateral thigh and on the proximal anterior thigh. The improvement in the thighs often reduces the extent of the thigh reduction surgery.

### Anatomical considerations

The skin-fat envelope of the thigh drapes over the underlying musculoskeletal core. It is less tightly adherent medially than antero-laterally. The entire thigh skin-fat envelope descends inferiorly in MWL patients. Although there is certain degree of vertical excess, most of the excess is horizontal in these thighs. As the medial adherence is not strong, the tissues in this area descend the maximum giving the impression that it is vertical rather than horizontal excess. There occurs medial vertical descent of the horizontal excess.

Superficial lymphatic structures and the great saphenous vein and its branches require special attention while performing medial thigh lift. The lymphatics of the leg are primarily concentrated medially and lie deeper than the saphenous vein until they coalesce in the femoral triangle. Injury to the lymphatics here can lead to disabling lower limb oedema which is usually permanent. In patients with significant varicose vein problems, the saphenous vein may also be excised along with medial skin resection.

### Patient presentation

The extent and type of the deformity in the thigh of MWL patients is extremely variable. Some male patients never deposit much fat in the thighs and thus lower body lift alone may achieve the acceptable thigh contours. Others may present with loose circumferential skin-fat envelope. These patients should undergo an excisional procedure only after a lower body lift to lift the lateral, posterior and upper-anterior thigh. However, most female MWL patients demonstrate a minimally deflated thigh despite an overall excellent weight loss. These patients should undergo concomitant liposuction of the thighs with the lower body lift, in preparation for a thigh reduction procedure to be performed six months later. In a nutshell, in order to attain acceptable contours, the thighs should be deflated either by massive weight loss process, or surgically by liposuction before the final excisional procedure.

### Preoperative evaluation

The extent and location of the horizontal and vertical soft tissue excess should be assessed. It is vital to rule out the pre-existing lymphoedema and deep vein thrombosis. Any evidence of lymphoedema or significant venous problems should be considered as contraindications for thighplasty. If horizontal excision is being incorporated, it is important to evaluate the degree of the traction transmitted across the perineal junction to the labia majora. The lateral traction thus exerted on the labia majora with subsequent exposure of the labia minora has often lead to medico legal actions against the surgeons.

### Marking and techniques

In MWL patient, the skin laxity can be quite severe and often extends down to and even below the knee. The traditional medial thigh lift techniques are marred by the problems of inferior wound migration, lateral traction deformity of vulva/ labia majora, widening of the scars and early recurrence of ptosis.[36] The Lockwood's classic horizontal resection and fascial anchoring technique for medial thigh lift designed to prevent labial spreading and migration of the perineal scar allows a more stable long-term result.[37,38] This technique has its *vector of pull vertically* toward the groin. However, the horizontal laxity seen in most MWL patients cannot be completely addressed with the classic vertical pull. Moreover, it also cannot address the middle and distal third of the thigh.

Rejuvenation of the medial thigh requires removal of the fat as well as excision and redraping of the entire medial thigh skin. The technique of vertical medial thigh lift incorporates the excision of the vertically oriented wedge of the excess skin located on the medial aspect of the thigh. It uses both an anterior and posterior *horizontal vector* while totally eliminating the vertical vector/pull, to accomplish thigh contouring. The horizontal incision in the groin is limited to the correction of the ‘dog-ear’ and does not contribute to the actual lift of the medial thigh. There is no need to anchor the thigh flaps to the Colles' fascia. The vertical excision closes the thigh as a cylinder. The tension of wound closure gets distributed from the groin to the knee along the medial thigh. Moreover, the vertical wedge is situated posterior enough so that if a horizontal component is needed to eliminate vertical excess, it would not cause labial distortion. The placement of excision also minimizes the possibility of injury to lymphatic structures reducing the risk of lymphoedema.

Markings are done with the patient in lithotomy position to mark the perineal crease. The tissue is pinched at various points along the perineal crease. The point at which the pinch has minimal traction on the labia is marked. This would be the top of the ellipse to be excised. Now with the patient standing with knees 12 inches apart a vertical line is drawn on the medial aspect of thigh starting at the point previously made at the perineal crease. This line will be the centre of the ellipse to be excised. The double ellipse technique of marking is used to delineate the wedge to be excised. With the patient in lithotomy position, the wedge is liposuctioned to completely deflate the area. The resection is then performed using the segmental resection closure technique and the wound is closed in layers over the suction drain. The dog-ear at the superior end is removed by extending the incision transversely, posteriorly along the infragluteal crease.

## FACE AND NECK

The face and the neck in a MWL patient are usually tackled at the end. Due to sudden and massive weight loss following bariatric surgery, the volume loss often gives the face an emaciated and gaunt appearance. Excessive facial fat atrophy coupled with redundant skin and SMAS leads to loss of facial contours and elasticity. The goal in facial rejuvenation in MWL patient is to restore the facial and neck contours. Multiplaner rhytidectomy addresses both volume and skin laxity. Secondary procedures are often performed to correct the residual deformities.

## POTENTIAL COMPLICATIONS

Considering the overall medical status of a post-bariatric surgery MWL patient who has rapidly lost massive amount of weight, nutritional deficiencies, anaemia, co-morbid conditions, and the extent of prolonged surgeries performed in various stages to achieve body contouring, relatively high incidence of complications is not unjustified. The complication rates increase with BMI. General complications include wound dehiscence, seroma, infection, delayed healing, lymphocele / lymphorrhea, suture extrusion, asymmetry, thrombosis, nerve compressions, neuroma, sensory and/or motor loss and scarring. Complications specific to particular procedures that require special attention have been described in the techniques above.

The incidence of complications can be reduced by appropriate management of co-morbid conditions before, during and after surgery, correction of anaemia and nutritional deficiencies, selection of the most suitable procedure for individual patients, precise markings, meticulous surgery, absolute hemostasis, three point sutures to obliterate the dead space, meticulous multi-layered closure over drains, properly instituted postoperative care, supervised nursing and regular follow-up.

Detailed planning of each stage and discussion with the patient, informed consent, and sound education of the patient, relatives and the nursing care team also contribute a lot to the overall success.

Wound dehiscence immediately after surgery is secondary to inappropriate movement by the patient and/or staff and is most often seen in the lower back. Patients should be fully alert and awake prior to initiation of any movements. Seromas may lead to delayed dehiscence and can result in chronic wounds with delay in healing. They are treated by serial aspirations, injections with a sclerosing agent (Povidone iodine, dicloxacillin) or exteriorizing the seroma cavity by making a small opening through the scar into the cavity, placing a Penrose drain and leaving it in place till the cavity fills itself and stops draining. Fibrin tissue sealants have been used to decrease the size and frequency of seromas.[39] Reports have also been published suggesting the role of ultrasonically activated Harmonic scalpel in the reduction of seromas in circumferential body lift procedures.[40]

Two issues that often lead to litigations are vulval distortion/ labial distraction after thighplasty, and brachioplasty closure. They have already been discussed under the respective sections above.

## SAFETY CONSIDERATIONS

The number of patients undergoing body contouring surgery following MWL has increased substantially in the past decade. Patient safety should be the topmost priority of the body-contouring surgeon simultaneously striving to achieve the desired aesthetic outcome. Comprehensive informed consents separate for each contouring procedure are mandatory.

### Medical status

Special attention should be paid to the current medical status of the patient and the accompanying co-morbid conditions like diabetes, hypertension etc. Weight stabilization must be ensured considering the co-morbidities and complexity of various diseases affecting the MWL patients, complete lab investigations, psychiatric evaluation, cardio-pulmonary clearance, PAC by experienced anaesthesiology team should be a routine. Smoking habit should be documented and should be strongly discouraged. Anaemia and nutritional deficiencies should be suitably corrected preoperatively. Patients should be informed about the possibility of intraoperative blood transfusions.

### Anesthesia concerns

MWL patients are difficult to intubate and may require fibreoptic intubation and neck lines. Many suffer from gastro oesophageal reflux and obstructive sleep apnoeas. These patients have increased risk of aspiration. Continuous monitoring must continue in post-op period. Large percentage of exposed skin for prolonged periods can lead to hypothermia. Use of warming air blankets, pre-warmed IV fluids, warming devices etc is recommended.

### Positioning during surgery

Patients frequently require supine, prone and lateral decubitus positions during surgery. This carries risk for neural and vascular compressions. Possible complications include vertebral artery occlusion, vision loss and nerve damage. Liberal use of soft pillows, gel mattresses and foam padding at nerve and bone prominences help prevent neuropraxia and skin necrosis. Regions to be cared for include occiput, orbit sockets, ears, cervical spine, shoulder blades, breast and nipples, sacrum, iliac crest and knees.

### Infection control

Consequent to nutritional deficiencies, diabetes, anaemia, impaired immune state and underlying bacterial and fungal skin infections, proper antibiotic prophylaxis for infection control is mandatory. For longer, multistage procedures, the risk of infections increases further. Topical broad spectrum soaps should be prescribed for twice daily bath for three days preoperatively. Injectable antibiotic should be administered one hour before surgery, during surgery and for at least 24h post surgery. Additionally, clipping of hairs rather than shaving reduces the chances of minor cuts and thorough scrubbing with 4% chlorhexidine gluconate solution helps disinfect the skin.

### DVT and pulmonary embolism prophylaxis

The risk of thrombosis in MWL patients undergoing body contouring is high because of extended operation time, the size of the wound area and the potential fat trauma. Contributing risk factors are use of oral contraceptives, pregnancy, advanced age, recent surgery, coagulopathies and prolonged immobilization. Thrombosis prophylaxis should include use of intra-operative compression treatment of lower legs, low molecular weight heparin, circulation promoting measures such as infusions of 2-3 litres of RL for dilution of circulating blood, postoperative beach chair position, early mobilization etc. Intermittent compression pumps may also be used as an alternative to compression stockings.

## CONCLUSIONS

The number of bariatric surgery procedures is on the increase in India. In the near future we are going to see large number of MWL patients who will require multiple body contouring procedures. The plastic surgery community in India has to prepare itself to effectively deal with these patients.
